# Flexible Manipulation of the Reflected Wavefront Using Acoustic Metasurface with Split Hollow Cuboid

**DOI:** 10.3390/ma15031189

**Published:** 2022-02-04

**Authors:** Limei Hao, Xi Chen, Xiaole Yan, Yujia Li, Li Zhang, You Xie, Shaofang Pang, Zhi Chen

**Affiliations:** 1 Department of Applied Physics, Xi’an University of Science and Technology, Xi’an 710054, China; chenxi290233@163.com (X.C.); yanxl@xust.edu.cn (X.Y.); L19940922yj@163.com (Y.L.); zl17732363928@163.com (L.Z.); xieyou@xust.edu.cn (Y.X.); pangshaofang@126.com (S.P.); 2 Department of Applied Physics, Northwestern Polytechnical University, Xi’an 710129, China

**Keywords:** acoustic metasurface, anomalous reflection, comprehensive manipulation, ultrathin

## Abstract

This work proposes a method for actively constructing acoustic metasurface (AMS) based on the split hollow cuboid (SHC) structure of local resonance, with the designed AMS flexibly manipulating the direction of reflected acoustic waves at a given frequency range. The AMS was obtained by precisely adjusting any one or two types of structural parameters of the SHC unit, which included the diameter of the split hole, the length, width, height, and shell thickness of the SHC. The simulation results showed that the AMS can flexibly manipulate the direction of the reflected acoustic waves, and the anomalous reflection angle obeys the generalized Snell’s law. Furthermore, among the five structural parameters, the AMS’s response frequency band is widest with the hole diameter and height, followed by the length and width, and narrowest with the shell thickness. It is worth noting that comprehensive manipulation of two parameters not only broadens the response frequency band, but also strengthens the effect of the anomalous reflection at the same response frequency. The subwavelength size of the AMS constructed with such a comprehensive method has the advantages of a small size, wide response band, simple preparation, and flexible modulation, and can be widely used in various fields, such as medical imaging and underwater stealth.

## 1. Introduction

The manipulation of wavefront distribution is a hotspot in physics and engineering technology. The emergence of metamaterials has brought new design ideas for wavefront manipulation. In 1996, Pendry et al. proposed the first artificial metamaterial composed of periodic structures which could achieve negative dielectric constant and negative permeability [1,2]. Based on the negative property, the wave can be manipulated and the metamaterials have important applications in infrared imaging [3], focus second-harmonic beams [4], meta-holograms [5], cloak [6], absorbers [7], and so on. Analogous to electromagnetic metamaterials, in 2000, Liu et al. presented the first artificial acoustic local resonance-type metamaterial which can achieve negative effective mass densities at a fixed frequency of 400 Hz [8,9]. Since then, acoustic metamaterials have been rapidly developed.

In 2006, Fang et al. proposed acoustic metamaterials with a negative effective bulk modulus, which consisted of a one-dimensional Helmholtz resonator structure [10]. In 2008, Yang et al. fabricated and characterized a membrane-type metamaterial with a two-dimensional film mass block structure in their experiments, achieving negative mass density [11]. Zhu et al. demonstrated a three-dimensional holey-structured metamaterial that achieved acoustic imaging [12]. Furthermore, metamaterial exhibit a unique property, only with difficulty, found in natural materials such as reflection and refraction [13], negative reflection [14], carpet cloaking [15], focusing [16], ultra-thin planar lenses [17], and super absorption [18].

Due to the limitations of the high inherent loss of metamaterials and the difficulties in preparing metamaterials, Yu et al. originally proposed the concept of metasurface which can modulate the direction of wavefront distribution, and no longer obeys the classical Snell’s law, satisfying the generalized Snell’s law [19,20]. Metasurfaces are passive phase-controlled arrays based on the generalized Snell’s law, and have a much smaller thickness than the working wavelength. In 2014, Cummer et al. [21] proposed an acoustic metasurface (AMS) with tapered labyrinthine units and AMS constructed by changing the helix angle of the labyrinth. The recent results indicated that AMS can obtain anomalous refraction phenomena. Tang et al. [22] also used the coiling structure unit to product AMS, and achieved the anomalous refraction modulation. Jiang et al. [23] demonstrated that all-angle negative refraction can be attained by altering the coiling angle of the labyrinthine structures from the standpoint of simulation and experiment. Zhao et al. [24] designed ultrathin acoustic metasurfaces based on membrane and cavity structures, and anomalous modulation of the transmitted acoustic waves was achieved. The AMS can not only manipulate refracted acoustic waves, but also modulate reflected acoustic waves. Li et al. [25] designed an ultrathin planar acoustic metasurface with the coiling up space, altered the phase at the interface by changing the acoustic path of acoustic waves propagating, and subsequently found anomalous reflection. An acoustic gradient metasurface was established with coiling-up space units, and anomalous reflections at all-angle incidence were obtained [26]. Zhao and Zhai [27] designed the AMS with a structure unit with a split cavity and an annular split cavity, and the anomalous modulation of the reflected acoustic waves was attained by changing the rotation angle of the inner split cavity. Liang et al. [28] designed a reflected acoustic hypersurface with Schroeder diffusers based on a subwavelength local resonance unit.

In all these reported works, the main problem with the local resonance AMS is that the prepared method is relatively single, and the frequency range of adjustment of the reflected wavefront distribution is relatively fixed, which makes it difficult to satisfy the engineering design needs at the various working frequency bands.

Our research groups confirmed that the resonance frequency was changed by adjusting the geometric size of the single-layer or multilayer SHS or SHC structures, and their precise analytical formulas were given [29,30]. In this work, the controllable AMS with ten split hollow cuboids (SHCs) was constructed by adjusting the different geometric parameters. The influence of the hole diameter, length, width, height, and shell thickness of the SHC on the reflected acoustic wavefront distribution were investigated in detail. Finally, comprehensive manipulation of two structural parameters was also studied.

## 2. Model and Simulation

**Figure 1:**
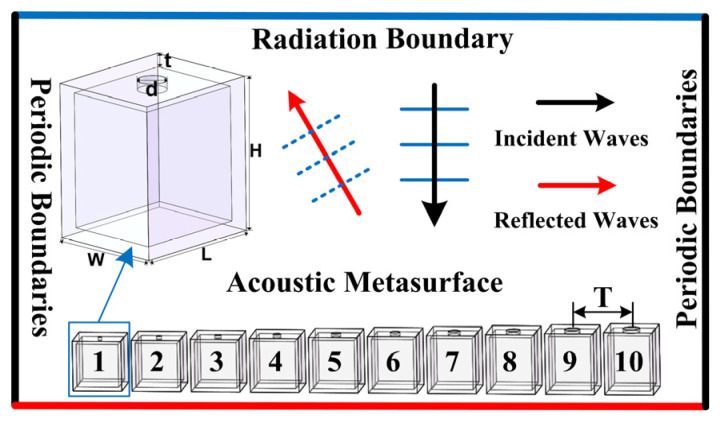
Cross section view of the acoustic metasurface (AMS) with a periodic boundary condition in the simulation environment.

COMSOL Multiphysics with the finite element method (FEM) was used to establish and simulate AMS, where the pressure acoustics module was selected to estimate. AMS is composed of ten SHCs structure units with different geometrical parameters, and the detailed simulation condition was set as shown in Figure 1. The four side faces were set as a periodic boundary. The top face was a radiation boundary with an 1 Pa planar acoustic wave, which was normally incident on the AMS. The bottom transmitted face was set as a hard boundary. In the simulation, the SHC was selected as a polylactic acid, and its modulus and density was $3\times 10^{9}$ Pa, 1250 kg/m^3^, respectively. Further, the inner part of the waveguide and the cavity in the SHCs were provided with air medium, and its density was 1.29 kg/m^3^, with the speed of sound in air as 343 m/s. The whole simulation domain was meshed by the tetrahedron based on the face mesh. The maximum element size was 1300(m/s)/f_max_/15 for solid domain meshing, and 343(m/s)/f_max_/4 for fluid domain meshing. The model was calculated in the frequency domain.

When the acoustic wave was incident perpendicularly to the split hole of SHC, the SHC can be regarded as an acoustic Helmholtz resonator. According to the principle of sound-force analogy, SHC can be compared to a single spring oscillator system. Furthermore, the SHC cavity was equivalent to the spring stiffness coefficient ($K_{m}$), and the opening was equivalent to the object mass ($M_{m}$). As a result, the values were as follows:

(1) $$K_{m}=\frac{\rho_{0}c_{0}^{2}}{V},M_{m}=\frac{\rho_{0}l}{S}$$

where $\rho_{0}$ is the density of air; $c_{0}$ is the sound speed in air; *S* is the cross-sectional area of the hole; the volume of the cavity is calculated by V=(L−2t)(W−2t)(H−2t); the effective length of the hole is calculated by $l=t+0.305d+\frac{4}{3\pi }d$; *d*, *L*, *W*, *H*, and *t* is the diameter of the split hole, length, width, height and shell thickness of the SHC structure unit, respectively, as shown in inset of Figure 1. Furthermore, we primarily set *L* = *W* = *H* = 20 mm, *d* = 6 mm, and *t* = 0.5 mm.

Thus, the resonant frequency was deduced as follows:

(2) $$f=\frac{\sqrt{K_{m}/M_{m}}}{2\pi }=\frac{c_{0}d}{4\pi }\sqrt{\frac{\pi }{\left(L-2t)(W-2t)(H-2t)(t+0.305d+\frac{4}{3\pi }d\right)}}$$

It is well known that the local resonance of an SHC structure unit occurs around the resonant frequency, and its phase around the resonant frequency will exhibit a significant torsion [29], which is helpful in building the AMS with a reflection phase gradient. Meanwhile, Equation (2) shows that the resonant frequency of the SHC is related to five structural parameters: the diameter of the split hole, the length, width, height, and shell thickness.

Therefore, there are four steps in the design of the AMS. First, under the condition of the needed response frequency band and the angle range of the anomalous reflection, the phase gradient was roughly calculated according to the generalized Snell’s law, and then the minimum period was fixed. The ideal size of a certain varying structural parameter was estimated, and then this ideal parameter was substituted into Equation (2) to deduce the other structural parameters. It should be noted that the hole diameter cannot be selected as a too large of a value and the thickness met the subwavelength size. At the same time, the structural parameters to be changed can be one or two or more structural parameters. Second, the relationship of the needed varied geometric parameters and the reflection phase was scanned parametrically with COMSOL (version 5.1), and then the phase modulation map was drawn. The geometric parameters of ten SHCs were precisely determined from the plotted reflection phase modulation map, resulting in phase shifts that covered 2π with an equal step of π/5 at the desired center frequency. Third, parameter sizes of ten determined SHS were exported to Allcct software (version: Allcct-YinKe-A1.0), print format was set in Allcct software, and then the file was saved to an SD card. The SD card was inserted into the 3D printer (Allcct-YinKe 200, Wuhan Allcct Co., Ltd., Wuhan, China), and then ten SHCs were printed. Finally, AMS with the positive phase gradients were constructed by arranging ten SHCs according to the reflected phase from small to large (see inset of Figure 1), and then the flexible modulation of the reflected acoustic waves were achieved in the desired frequency band. Furthermore, the central distance between two adjacent SHCs was denoted as the AMS period (T). The phase gradient of the AMS was closely related to the period, and its calculation formula was π/5T.

Here, it should be noted that when the selected parameter cannot meet the requirements of the reflection phase change within the required frequency range, or the geometric size difference of the ten SHCs is too large, it is necessary to estimate the varied structural parameter again, or comprehensively manipulate it with two or more structural parameters.

## 3. Results and Discussion

The generalized Snell’s law is $\sin(\theta_{r})-\sin(\theta_{i})=(c/2\pi n_{i}f)(d\varphi /dx)$, where $\theta_{i}$ is the angle of incidence, $\theta_{r}$ is the angle of reflection, f is frequency, $n_{i}$ is refractive index, and dφ/dx is the phase gradient in the direction of x. Based on the generalized Snell’s law, when the acoustic wave is vertically incident to a surface with the phase gradient of 0 rad/mm, the reflected acoustic wave is emitted vertically. When the acoustic wave is vertically incident to a metasurface with the phase gradient, the reflected acoustic wave is emitted in a nonvertical direction, resulting in an anomalous reflection.

Therefore, the flexible manipulations of the acoustic wavefront distribution are discussed with the gradient AMS composed of ten SHCs by adjusting one or two types of geometric parameters based on the generalized Snell’s law.

### 3.1. Effect of the Hole Diameter

**Figure 2:**
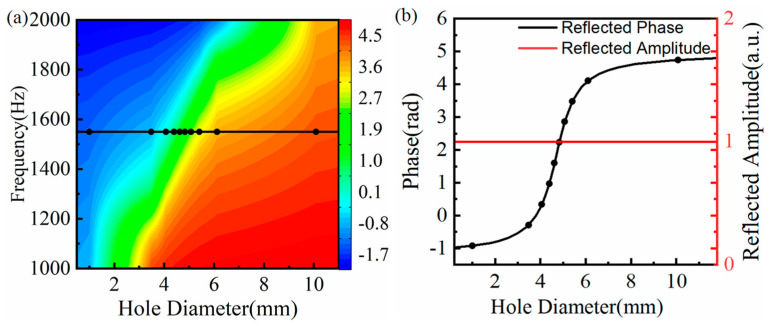
(**a**) The reflection phase modulation map of split hollow cuboids (SHCs) with different split hole diameters, where the corresponding frequency of the black curve is the designed center frequency; and (**b**) the reflection phase and reflection amplitude of the SHC with the different split hole diameters at the frequency of 1550 Hz, where the corresponding diameter of the black circle symbols is chosen as the split hole diameter of ten SHCs.

In this section, when the side length and thickness of ten SHCs structural units were fixed as 20 mm and 0.5 mm, the diameter of the spilt hole varied from 1 mm to 12 mm. Figure 2a plots the phase modulation map of the SHC structural unit. Considering the effect of the hole diameters on the SHC structure unit, the center frequency was set to 1550 Hz (see the black curve in Figure 2a). Furthermore, Figure 2b depicts the relationship curve between the reflection phase and the hole diameter at the center frequency of 1550 Hz. It is shown that the reflection amplitude are all close to one, and the phase shifts cover 2π by ten SHCs structure units with an equal step of π/5. Thus, the AMS with positive phase gradients was constructed by assembling ten SHCs with the varied diameter of the split hole from small to large, according to the parameters of ten black circle symbols in Figure 2b, and its thickness is only λ/9.

**Figure 3:**
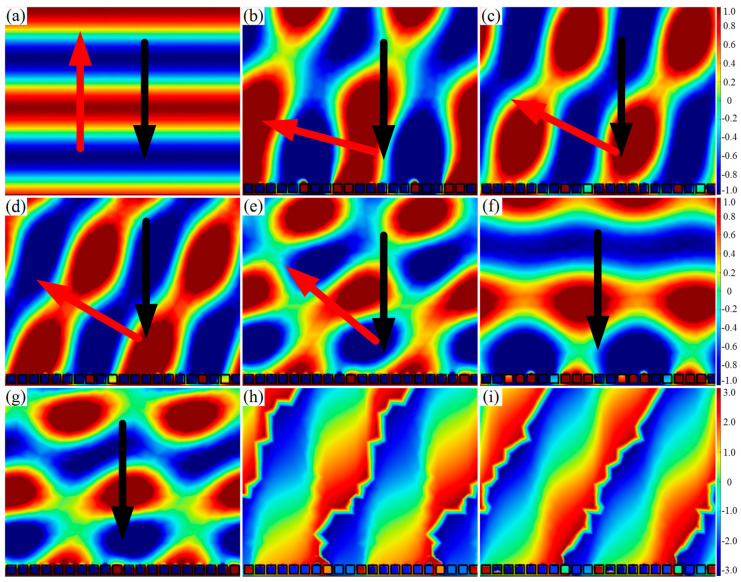
AMS of ten SHCs with different hole diameters. (**a**) Reflected acoustic pressure field distribution at the frequency of 1550 Hz for 0 rad/mm; (**b**–**g**) reflected acoustic pressure field distribution at 1425 Hz, 1550 Hz, 1590 Hz, 1790 Hz, 1390 Hz, and 1850 Hz for π/125 rad/mm, respectively; and (**h**,**i**) phase distribution at 1550 Hz and 1590 Hz.

Figure 3a is the reflected acoustic pressure field distribution of the surface with the phase gradient of 0 rad/mm. As seen from Figure 3a, when the acoustic wave was perpendicularly incident on the surface, the reflect wave was also perpendicularly emitted, which follows the classic Snell’s law. The acoustic wave was perpendicularly incident onto the AMS with the phase gradient of π/125 rad/mm, and the reflected acoustic pressure field distribution is depicted in Figure 3b–g. It is found from Figure 3b–e that the anomalous reflection occurred at frequencies of 1425 Hz, 1550 Hz, 1590 Hz, and 1790 Hz. Meanwhile, the reflection angles were measured as 74.8°, 63.3°, 60.0°, and 50.3°, respectively, and these angles agree with the theoretical angles of 74.3°, 62.3°, 59.6°, and 50.0° based on the generalized Snell’s law. As shown in Figure 3f–g, both of the reflected acoustic pressure field became confused and no longer satisfied Snell’s law when the frequencies were 1390 Hz (below 1425 Hz) and 1850Hz (above 1790 Hz). Thus, AMS with the phase gradient of π/125 rad/mm achieved flexible modulation of the reflected acoustic wavefront distribution with a bandwidth of 365 Hz at a frequency range from 1425 Hz to 1790 Hz. Table 1 shows the reflection phase shifts of ten SHCs at frequencies of 1425 Hz, 1550 Hz, and 1790 Hz, respectively. It can be seen from Table 1 that the reflection phase shifts did not cover 2π at 1425 Hz and 1790 Hz. The reflection phase changed more drastically in the first several SHC units at 1425 Hz, whereas it changed more drastically in the last several SHC units at 1790 Hz. Therefore, the reflection phase has an effect on the response frequency band. In addition, comparing Figure 3h with Figure 3i, the reflected phase distribution was more uniform and continuous at the frequency of 1590 Hz than that of 1550 Hz. Thus, the practical center frequency of AMS is 1590 Hz.

### 3.2. Effect of the Length

In order to investigate the effect of SHC length on the manipulation of the reflected acoustic wavefront distribution, the SHC geometric parameters were fixed as per Section 3.1, the hole diameter was 6 mm, and the SHC length varied from 8 mm to 35 mm.

**Figure 4:**
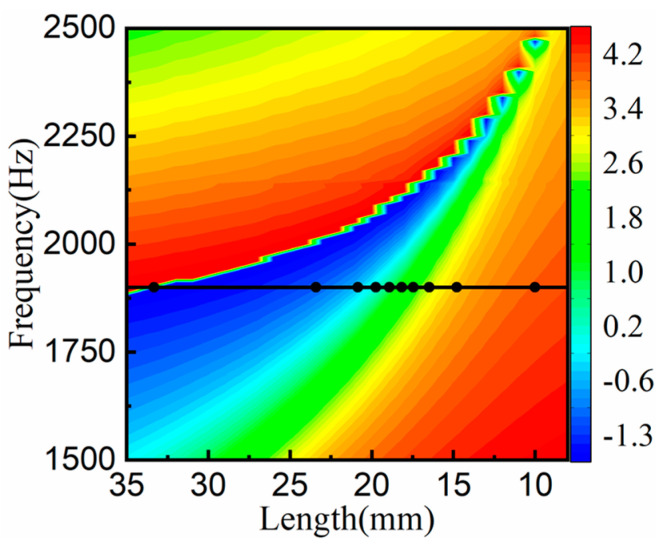
The reflection phase manipulation map of SHCs with different lengths, where the corresponding frequency of the black curve is the designed center frequency and the corresponding length of the black circle symbols is selected as the length of ten SHCs.

Figure 4 depicts the phase modulation map of the SHC structural unit. As demonstrated in Figure 4, the reflected phase shifts between SHCs units were more uniform around 1900 Hz, and so the center frequency was chosen as 1900 Hz (see the black curve). The corresponding length of the black circle symbols in Figure 4 were selected as the lengths of the ten SHCs to cover the reflection phase shifts of 2π. The AMS with the phase gradient of π/122 rad/mm was constructed by arranging the ten SHCs of varying length from long to short, and the thickness of the AMS was λ/7.

**Figure 5:**
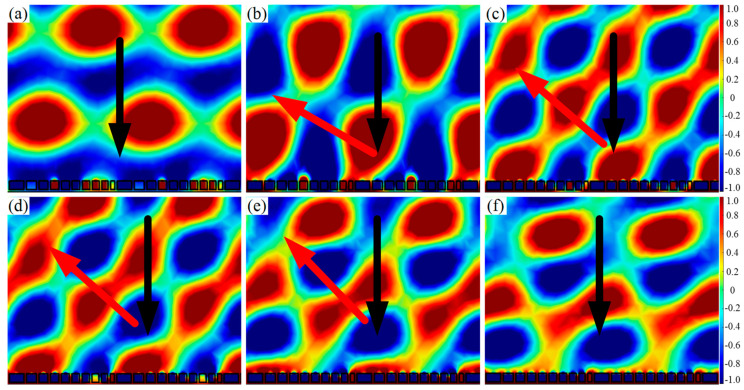
Reflected acoustic pressure field distribution of the AMS of SHCs with different lengths at the phase gradient of π/122 rad/mm. (**a**) 1625 Hz; (**b**) 1645 Hz with 60.1°; (**c**) 1875 Hz with 49.2°; (**d**) 1900 Hz with 48.3°; (**e**) 1950 Hz with 46.0°; and (**f**) 2000 Hz.

Figure 5 demonstrates the reflected acoustic pressure field distribution at different frequencies under the condition of a vertically incident acoustic wave. It is seen from Figure 5b–e that the anomalous reflection occurred at frequencies of 1645 Hz, 1875 Hz, 1900 Hz, and 1950 Hz, with measured reflected angles of 60.1°, 49.2°, 48.3°, and 46.0°, respectively. Meanwhile, according to the generalized Snell law, the corresponding theoretical angles were calculated with 59.1°, 48.8°, 48.0°, and 46.4°, and these angles consist with the corresponding measured angles. Therefore, AMS with the phase gradient of π/122 rad/mm may flexibly manipulate the reflected acoustic wavefront distribution with a bandwidth of 305 Hz at a frequency range of 1645 Hz to 1950 Hz. The reason for this phenomenon is that the response frequency band was affected by the volume of the SHC cavity, resulting from the change of the SHC length. In addition, comparing Figure 5c with Figure 5d, the reflected wavefront distribution at 1875 Hz was more continuous than that at 1900 Hz. Thus, the practical center frequency of AMS was 1875 Hz.

### 3.3. Effect of the Width

In order to master the effect of SHC width on the manipulation of the reflected acoustic wavefront distribution, other geometric parameters of SHCs were fixed as per Section 3.1, and the width of SHCs varied from 8 mm to 32 mm.

**Figure 6:**
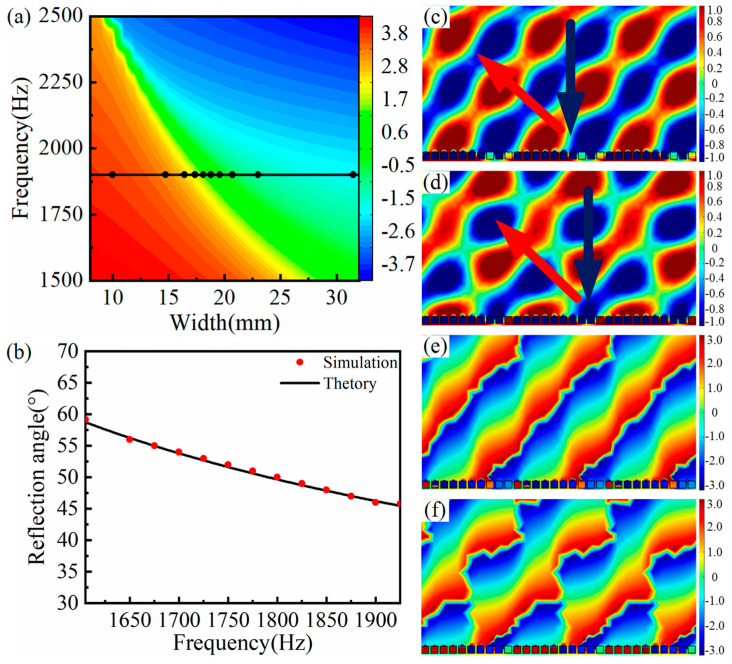
AMS of ten SHCs with different widths at the phase gradient of π/125 rad/mm. (**a**) The reflection phase modulation map, where the corresponding frequency of the black curve is the designed center frequency and the corresponding width of the black circle symbols is chosen as the width of ten SHCs; (**b**) the AMS relationship curve between frequency and the anomalous reflected angle; (**c**) and (**d**) reflected acoustic pressure field distribution at 1850 Hz and 1900 Hz; and (**e**) and (**f**) reflected phase distribution of corresponding (**c**) and (**d**), respectively.

Figure 6a shows the reflection phase modulation map of the SHC structure unit with different widths. Considering the requirement of the subwavelength scale, the center frequency was set as 1900 Hz, and the width of the corresponding black circle symbols was selected as the width of the ten SHCs (see Figure 6a). Thus, the AMS with the phase gradients of π/125 rad/mm was constructed by assembling ten SHCs with varying width from broad to narrow.

The relationship curve between frequency and the anomalous reflection angle of the AMS with the phase gradients of π/125 rad/mm is depicted in Figure 6b, where the black solid curve is the calculation curve of anomalous reflection angle based on the generalized Snell’s law, and the red circle symbols are the measured anomalous reflection angle from the reflected wavefront distribution. Figure 6b shows that the measured reflection angles coincide with the corresponding theoretical angles, and the reflection angle is inversely proportional to frequency. For example, when the corresponding lowest and maximum frequency of the anomalous reflection were 1605 Hz and 1925 Hz, the simulated reflection angles were measured as 59.2° and 45.8°, the theoretical angles were calculated as 58.7° and 45.5°, and they are in good agreement with each other. Thus, AMS obtained a flexible modulation of the reflected acoustic wavefront distribution with a bandwidth of 320 Hz at a frequency range of 1605 Hz to 1925 Hz. In addition, similar to Section 3.2, it was found from Figure 6c–f that the reflected wavefront distribution and phase distribution at 1850 Hz were more uniform than those at 1900 Hz, implying that the practical center frequency of the AMS is 1850 Hz.

### 3.4. Effect of the Height

In order to investigate the effect of SHC height, the other geometric parameters of SHCs were fixed as per Section 3.1, and the height of the SHCs varied from 8 mm to 32 mm.

**Figure 7:**
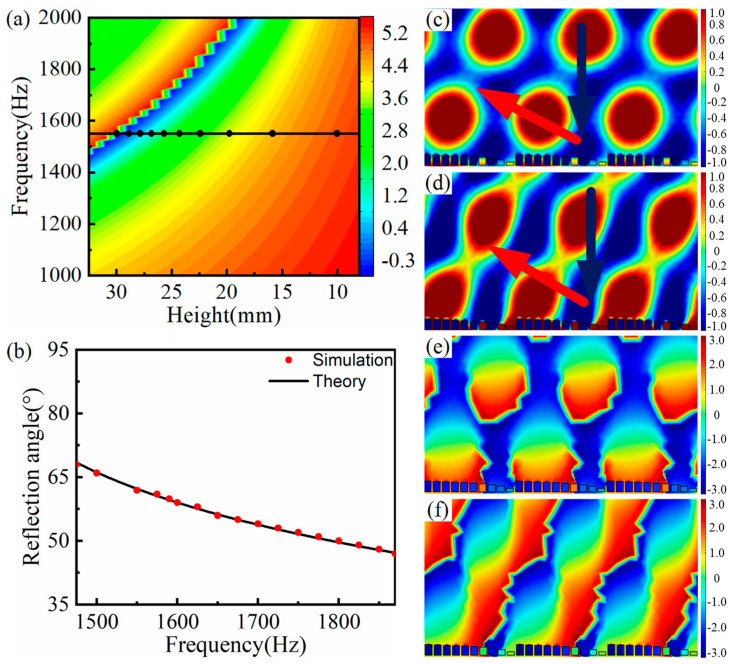
AMS of ten SHCs with different heights at the phase gradient of π/125 rad/mm. (**a**) The reflection phase modulation map, where the corresponding frequency of the black curve is the designed center frequency and the corresponding height of the black circle symbols is chosen as the height of ten SHCs; (**b**) the AMS relationship curve between frequency and the anomalous reflection angle; (**c**,**d**) reflected acoustic pressure field distribution at 1550 Hz and 1590 Hz; and (**e**,**f**) reflected phase distribution of corresponding (**c**) and (**d**), respectively.

Figure 7a plots the reflection phase modulation map of the SHC structure unit with varying height. Considering the geometric differences among ten SHCs units, the center frequency was set as 1550 Hz and the corresponding height of the black circle symbols were selected as the height of ten SHCs (see the Figure 7a). Thus, the AMS with the phase gradient of π/125 rad/mm was constructed by arranging ten SHCs with varying height from high to low.

The relationship curve between frequency and the anomalous reflection angle is shown in Figure 7b, where the red circle symbol represents the measured reflection angle from the reflect wavefront distribution, and the black solid curve represents the calculated curve using the generalized Snell’s law. As illustrated in Figure 7b, those measured angles were consistent with the corresponding theoretical angle. For example, with a central frequency of 1590 Hz, the theoretical and measured reflection angles were 59.6° and 59.9°, respectively, and they are in good agreement with each other. Thus, AMS attained variable modulation of the reflected acoustic wavefront distribution with a bandwidth of 395 Hz at a frequency range of 1475 Hz to 1870 Hz. In addition, similar to the previous section, it was deduced from the Figure 7c–f that the practical center frequency of the AMS was 1590 Hz, and this practical center frequency shift may be induced by the weak interaction between the SHCs units.

### 3.5. Effect of the Shell Thickness

**Figure 8:**
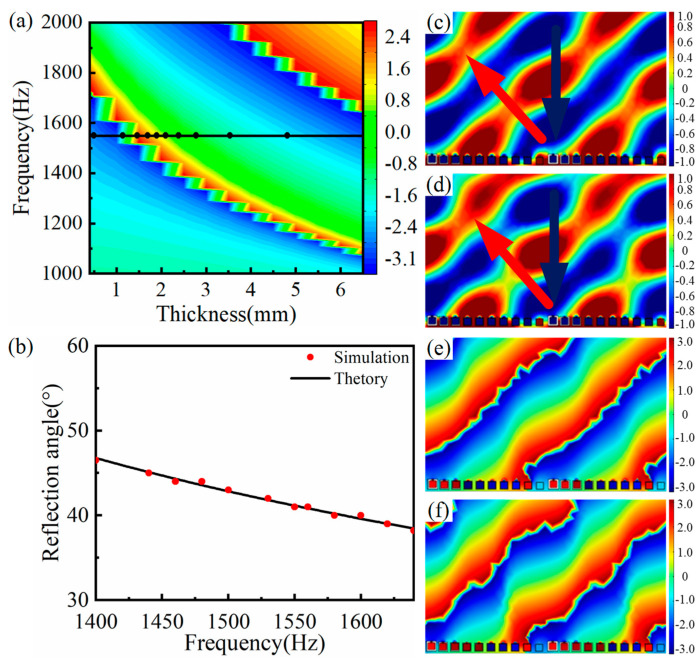
AMS of ten SHCs with different SHC shell thicknesses at the phase gradient of π/168 rad/mm. (**a**) The reflection phase modulation map, where the corresponding frequency of the black curve is the designed center frequency and the corresponding thickness of the black circle symbols is chosen as the thickness of ten SHCs; (**b**) the AMS relationship curve between frequency and the anomalous reflection angle; (**c**,**d**) reflected acoustic pressure field distribution at 1530 Hz and 1550 Hz; and (**e**) and (**f**) reflected phase distribution of corresponding (**c**) and (**d**), respectively.

In order to master the influence of the shell thickness of the SHC, other geometric parameters were fixed as the previous section, and the shell thickness varied from 0.5 mm to 6.5 mm. Figure 8a demonstrates the reflection phase modulation map of the SHC structure unit with varying shell thicknesses. Considering the requirement of the subwavelength scale, the center frequency was set to 1550 Hz and the corresponding thickness of the black circle symbols was selected as the thickness of the ten SHCs, as seen from Figure 8a. Thus, the AMS with the phase gradients of π/168 rad/mm was constructed by assembling ten SHCs of varying thickness from thick to thin.

The relationship curve between the frequency and anomalous reflection angle is depicted in Figure 8b, where the black curve represents the calculated theoretical anomalous reflection angle based on the generalized Snell’s law and the red circle symbol represents the measured anomalous reflection angle. It can be found from Figure 8b that those measured reflection angles were consistent with the corresponding theoretical angles. For example, when the minimum and maximum frequency was 1400 Hz and 1640 Hz, the calculated angles were 46.8° and 38.4°, whereas the measured reflection angles were 46.5° and 38.2°, all of which coincide with each other. Thus, AMS obtained the flexible modulation of the reflected acoustic wavefront distribution with a bandwidth of 240 Hz at a frequency range of 1400 Hz to 1640 Hz. It is thought that the change of the SHC shell thickness simultaneously varied the hole diameter and cavity volume, resulting in the narrower response frequency band.

In addition, similar to Section 3.4, it was deduced from Figure 8c–f that the practical center frequency of the AMS was 1530 Hz. Meanwhile, the center frequency shifted from 1550 Hz to 1530 Hz, which resulted from the weaker interactions between the SHCs units.

### 3.6. Comprehensive Manipulation of Two Parameters

**Figure 9:**
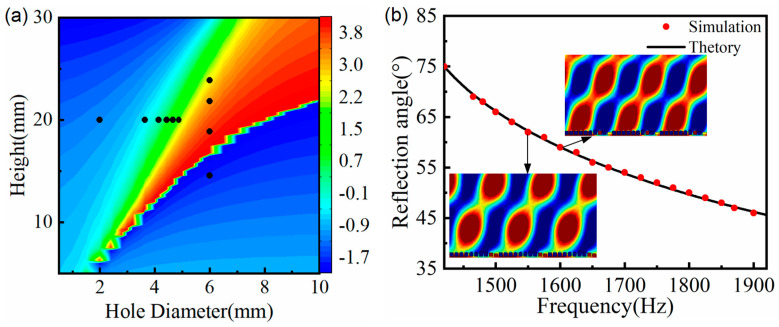
AMS of ten SHCs with two different parameters at the phase gradient of π/125 rad/mm (**a**) The reflection phase modulation map of SHC unit with two different parameters; and (**b**) the AMS relationship curve between frequency and the anomalous reflection angle.

In order to broaden the response frequency band, a comprehensive manipulation method was applied by adjusting two geometrical parameters of SHC. Both the hole diameter and height of SHC varied, but the other geometric parameters of SHC were fixed as per the previous section. Figure 9a plots the reflected phase modulation map of the SHC unit with various hole diameters and heights. In order to discuss the difference between the comprehensive and single manipulations, the center frequency was still 1550 Hz, which was the same as the center frequency of Section 3.1 and Section 3.2. The AMS was constructed by arranging six SHCs units with varying hole diameters and four SHCs units with varying heights.

Figure 9b demonstrates the relationship curve between the frequency and anomalous reflection angle at the frequency range of 1420 Hz to 1920 Hz, where the red circle symbol is the measured reflection angle, and the black solid curve is the calculated curve using the generalized Snell’s law. As seen from Figure 9b, the measured reflection angles accorded with the corresponding theoretical angles. For example, when the minimum and maximum frequency were 1420 Hz and 1920 Hz, the calculated theoretical angles were 75.1° and 45.6°, respectively, whereas the measured reflection angles were 75.0° and 46.0°, which is in good agreement with theoretical angles. Thus, AMS obtained the flexible modulation of the reflected acoustic wavefront distribution with a bandwidth of 500 Hz at the frequency range of 1420 Hz to 1920 Hz. Furthermore, as mentioned in the previous section, the practical center frequency of AMS was 1600 Hz, and it was the reason that the reflected wavefront distribution and phase distribution were more uniform and continuous at 1600 Hz than at 1550 Hz (see the two illustrations in Figure 9b).

**Figure 10:**
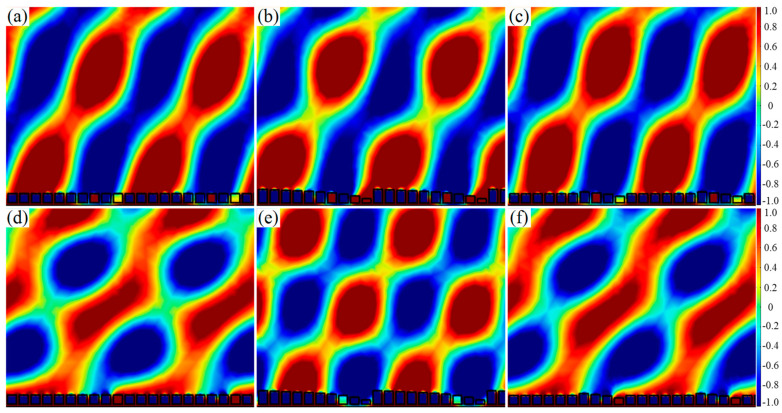
Reflected wavefront distribution of AMS of ten SHCs with varying parameters at the phase gradient of π/125 rad/mm. (**a**–**c**) the various hole diameters, various heights and various hole diameters and heights at 1590 Hz, respectively; and (**d**–**f**) various hole diameters and heights at 1725 Hz, respectively.

It is worth noting that the reflected acoustic wavefront distribution of the AMS designed by the two comprehensive modulations was more continuous than that designed by the single parameter modulation, as shown in Figure 10a–f. Therefore, the comprehensive manipulation not only broadened the response frequency band, but also improved the reflected acoustic wavefront distribution. It was mainly because the comprehensive manipulation reduced the geometric difference of the ten SHCs. 

**Figure 11:**
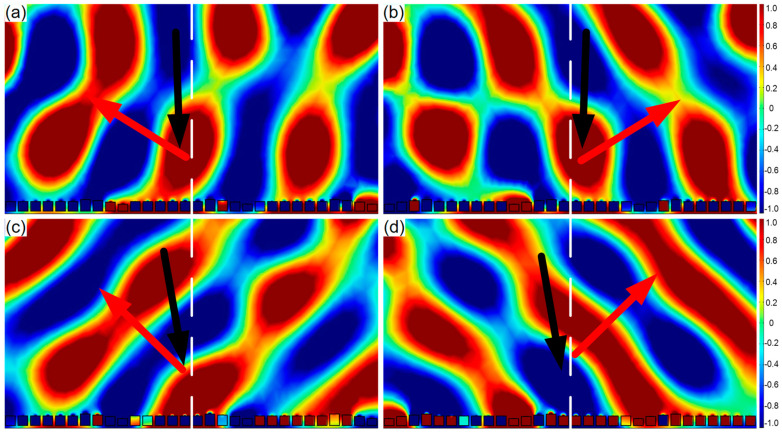
Reflected wavefront distribution of AMS of ten SHCs with varying parameters at 1550 Hz. (**a**) The phase gradient of π/125 rad/mm and the incident angle of −2°; (**b**) the phase gradient of −π/125 rad/mm and the incident angle of −2°; (**c**) the phase gradient of π/125 rad/mm and the incident angle of −10°; and (**d**) the phase gradient of −π/125 rad/mm and the incident angle of 10°.

According to the traditional Snell’s law, the incident wave and the reflected wave are divided on both sides of the normal line, and the reflected angle equals to the incident angle. The above designed AMS breaks this traditional law, and its reflection behavior obeys the generalized Snell’s law. Furthermore, two types of AMS were constructed by ten SHCs with the above-determined structural parameter. One type was AMS with a phase gradient of π/125 rad/mm from smallest to largest; the other type was AMS with a phase gradient of −π/125 rad/mm from largest to smallest. Their reflected wavefront distribution of AMS with a certain incident angle at 1550 Hz are shown in Figure 11. It can be seen from Figure 11a–c that both of oblique incident wave and the reflected wave are on the same side of the normal line, realizing the negative anomalous reflection. On the contrary, as illustrated in Figure 11d, the oblique incident wave and the reflected wave are on two sides of the normal line, realizing the positive anomalous reflection. It is worth noting that both the positive and negative anomalous reflection angles satisfied the generalized Snell’s law. 

Therefore, the flexible modulation of the reflected wavefront distribution can be realized by adjusting the positive and negative phase gradient, incident angle, incident direction, and response frequency.

## 4. Conclusions

In summary, this work achieved the flexible manipulation of the reflected wave direction by constructing gradient-type AMS by adjusting any one or two types of parameters of SHC’s structure units, which included the hole diameter, length, width, height and shell thickness. The results showed that the AMS phase shifts cover 2π with an equal step of π/5. The anomalous reflection occurred when the acoustic wave was vertically incident on the AMS, and these reflection angles satisfied the generalized Snell’s law. Meanwhile, the response frequency band of the anomalous reflection for the AMS with only tuning the hole diameter, length, width, height, or shell thickness was 365 Hz, 305 Hz, 320 Hz, 395 Hz, and 240 Hz, respectively. Furthermore, the comprehensive manipulation of two parameters not only broadened the response frequency band to 500 Hz, but also improved the anomalous reflected wavefront distribution at the same response frequency. Therefore, based on the method of comprehensive manipulation, the anomalous reflection can be actively obtained in any given frequency with the AMS by precisely changing one or two types of the SHC’s geometric parameters under the condition of meeting engineering requirements. The gradient-type AMS has promising applications in medical imaging, warship cloaking, beam control equipment, and so on.

## Figures and Tables

**Table 1:** The reflection phase of ten SHCs.

Frequency (Hz)	Serial Number									
	1	2	3	4	5	6	7	8	9	10
1425	−0.78	0.27	1.64	2.64	3.22	3.61	3.91	4.19	4.50	4.89
1550	−0.92	−0.29	0.33	0.96	1.60	2.23	2.85	3.48	4.11	4.74
1790	−1.26	−0.98	−0.81	−0.66	−0.51	−0.34	−0.07	0.53	2.47	4.42

## Data Availability

Not applicable.
